# Diastasis of the Pubic Symphysis Without Fall While Horseback Riding: A Case Report

**DOI:** 10.7759/cureus.72988

**Published:** 2024-11-04

**Authors:** Dimitrios Giotis, Christos Konstantinidis, Sotiris Plakoutsis, Dimitrios Vardakas, Vasileios Panagiotopoulos

**Affiliations:** 1 Orthopaedic Department, General Hospital of Ioannina G. Hatzikosta, Ioannina, GRC

**Keywords:** diastasis, fall, horse-riding, pelvic ring disruptions, pubic symphysis

## Abstract

The purpose of this study was to demonstrate a rare case of pelvic ring injury in a healthy man without a history of high energy damage. A 43-year-old man presented to the emergency with local pain in pubic symphysis and difficulty walking after horseback riding. The patient did not report any fall or injury during this recreational activity, and apart from tachycardia, he was hemodynamically stable with normal blood pressure. Additionally, no deficit of neurological function was observed. The radiological imaging demonstrated an injury of the pelvic ring APC II with a diastasis of pubic symphysis of 3.6 cm. After a temporary stabilization with a pelvic binder, a computed tomography scan was also executed. A closed reduction and stabilization of the pelvic ring with supraacetabular external fixation with two 6 mm pins was performed. Postoperatively, the diastasis of the pubic symphysis was reduced to 1.5 cm. The patient remained in bed for four weeks, and afterward, gradual mobilization with partial weight bearing was allowed with crutches. The external fixation was removed 10 weeks postoperatively, and he fully returned to his pre-injury activities without any discomfort four months after the injury. Pelvic ring injuries in young patients without high-energy injuries are extremely rare and might be misdiagnosed. As presented in this case, the sudden onset of pain in pubic symphysis, combined with difficulty walking after a similar low-energy task, should not be overlooked for pelvic injury.

## Introduction

Pelvic ring disruptions, including pubic symphysis diastasis and open-book pelvic fractures, are typically related to a high-velocity force, such as vehicle collisions, falls from a height, or crush injuries [1]. In cases of posterior instability, the role of early anatomical reduction, restoration of structural integrity, and immobilization of the pelvic ring is critical in order to control haemorrhage and haemodynamic instability [2].

Diastasis of the pubic symphysis is considered as a gap greater than 10 mm and has been reported in 15-24% of pelvic fractures, often associated with other pelvic ring injuries [1,3,4]. Apart from trauma, other secondary causes might involve pregnancy, symphysitis following radiotherapy, or several congenital and metabolic abnormalities [5]. Treatment options for pubic symphysis diastasis might include non-operative treatment with or without the application of pelvic binder, anterior external fixation with or without sacroiliac screw fixation, and anterior internal fixation with plate and screws, amongst others [1,2,5].

Various injuries are associated with horse riding accidents, not only at an amateur level but also at a professional level. Especially pelvic injuries after horse-related activities can be life-threatening, requiring emergency resuscitation [6]. In the vast majority of cases, the trauma mechanism involves a fall from a horse or a kick by a horse [7]. However, sporadic cases of pubic symphysis diastasis have been reported in the literature due to low-energy distraction that restricted the lesion to the anterior ring after horseback riding [8,9]. In these cases, the horse riders land astride the saddle's pommel violently after being thrown vertically when the horse is bucked [8,9].

The aim of the study is to present a rare case of a young, active man suffering a diastasis of the pubic symphysis after horseback riding without reporting any traumatic condition. We also highlight the clinical evaluation that was performed and the necessary management steps that contributed to a successful outcome.

This article was previously presented as a meeting abstract at the XXXVI World Congress of Sports Medicine on September 23-26, 2021.

## Case presentation

A 43-year-old recreational male horse rider, height: 1.81 m, weight: 88 kg, BMI (Body mass index): 26.86 kg/m^2^, presented to the Emergency Department of our Hospital 24 hours after horseback riding for 90 minutes, complaining of local pain in pubic symphysis and difficulty walking. He did not report any fall or injury during horse riding. Regarding the medical history, no chronic diseases, surgical procedures, history of trauma over the last 12 months, or medications were reported. The patient was working as a hotel manager without participating in other sports activities.

During clinical evaluation it was observed that he was able to weight bear but with discomfort and the pain was alleviated when sitting. No sign of scrotal, perineal or suprapubic bruising was observed but tenderness was obvious over the anterior aspect of the pelvis. The patient reported no other complaints such as urogenital symptoms and no other clinical abnormalities on examination of hips and lumbar spine were found. Additionally, no deficit of neurological function was noted.

**Figure 1:**
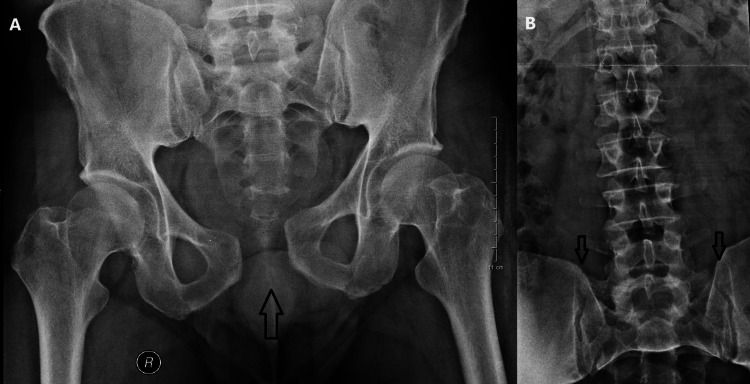
A) Initial anteroposterior radiograph of pelvis displaying wide diastasis of the pubic symphysis. B) Anteroposterior radiograph of the lumbar spine depicting no sign of sacroiliac joint disruption.

He was also hemodynamically stable on assessment, displaying normal blood pressure (115/78 mmHg), blood oxygen saturation (SpO₂: 96%), and respiratory rate (18 breaths per minute) but mild tachycardia (110 beats per minute). Catheterization of the bladder was performed without difficulty, and clear urine was obtained without the existence of blood. The radiologic evaluation revealed an injury of the pelvic ring APC II according to Young & Burgess classification, rotationally unstable and vertically stable, with a diastasis of pubic symphysis of 3.6 cm but with intact posterior sacroiliac joint ligaments (Figure 1) [10].

**Figure 2:**
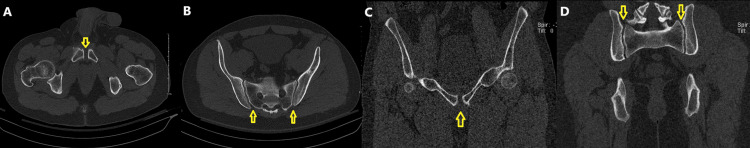
(A) CT transverse view highlighting the pubic symphysis. (B) CT transverse view highlighting the sacroiliac joints. (C) CT coronal view focusing on the pubic symphysis. (D) CT coronal view focusing on the sacroiliac joints.

After a temporary stabilization with a pelvic binder, a computed tomography scan (CT) was conducted. Its findings confirmed the type of pelvic injury with the presence of a small anterior haematoma and a 16 mm gap at the pubic symphysis as restored with the use of the pelvic sling. No evidence of sacroiliac joint disruption, sacral fracture, or other destabilizing posterior damage was noticed (Figure 2).

**Figure 3:**
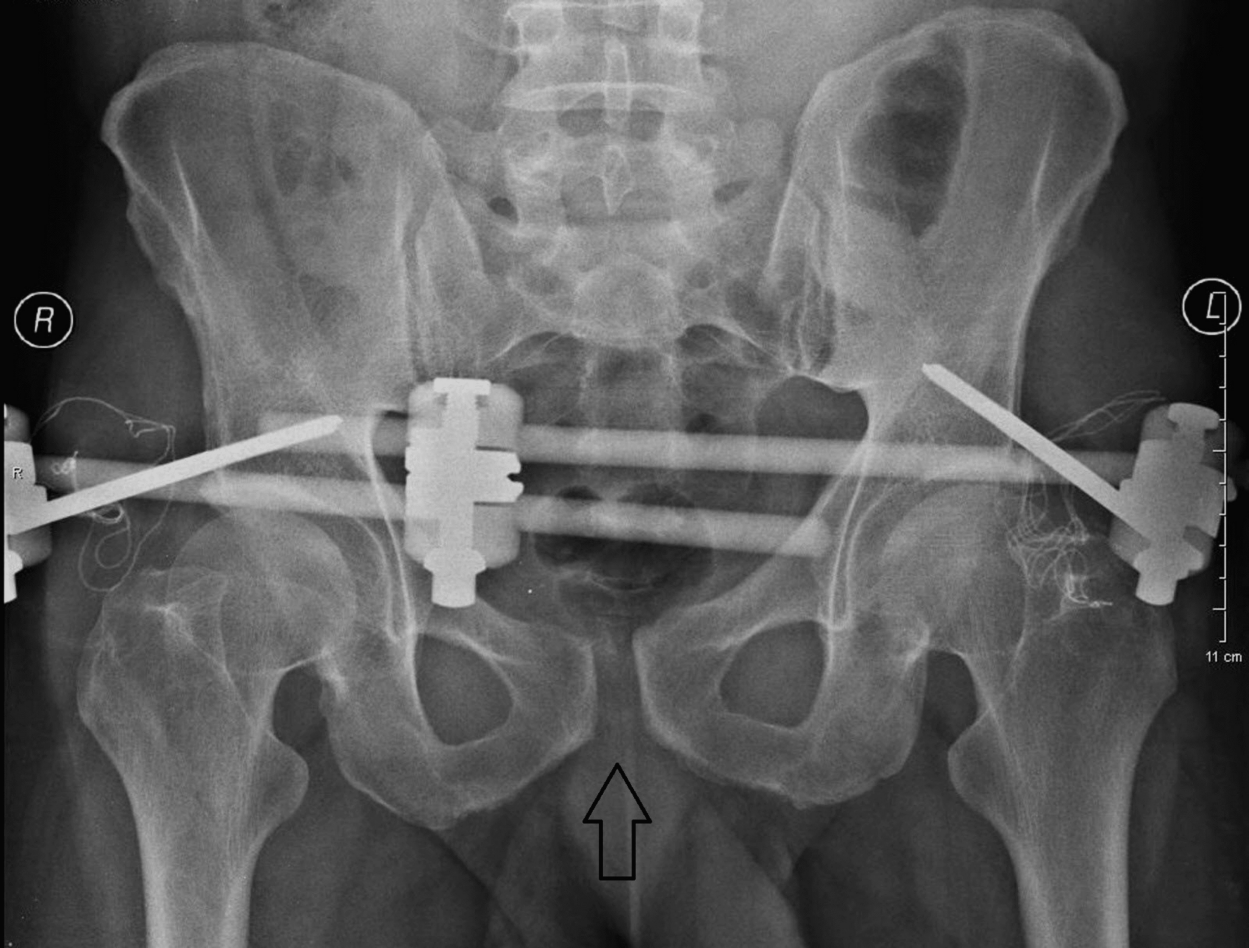
Anteroposterior X-ray after closed reduction and external fixation (First postoperative day).

The patient was taken to the theatre for closed reduction and stabilization of the pelvic ring with supraacetabular external fixation with two 6 mm pins. Postoperatively, the diastasis of the pubic symphysis was successfully reduced to 1.5 cm (Figure 3).

**Figure 4:**
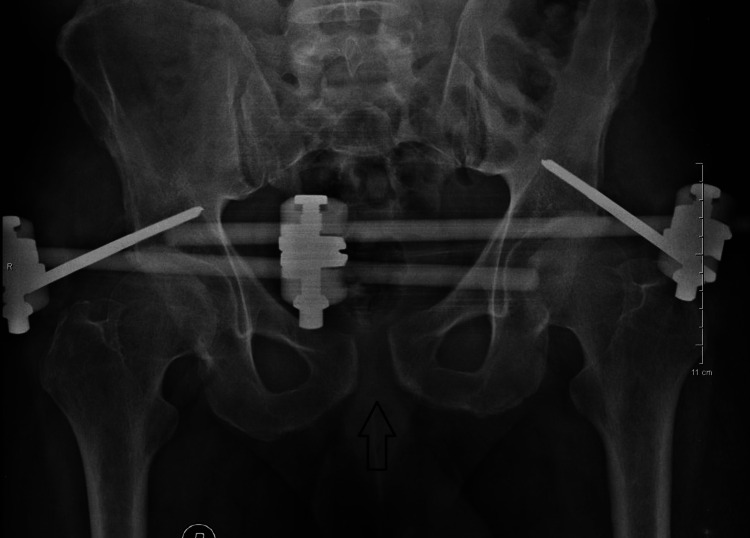
Anteroposterior X-ray after light widening of the pubic symphysis (seven weeks postoperatively).

One week later, he was discharged from the Hospital. He remained in bed for two weeks, and afterward, gradual mobilization with partial weight bearing was allowed with crutches. At six weeks, full weight bearing was recommended. One week later, the new X-ray revealed a light further widening of the pubic symphysis (1.8 cm gap) but without any accompanying symptomatology, and thus, the external fixation was removed only at 10 weeks postoperatively (Figure 4).

**Figure 5:**
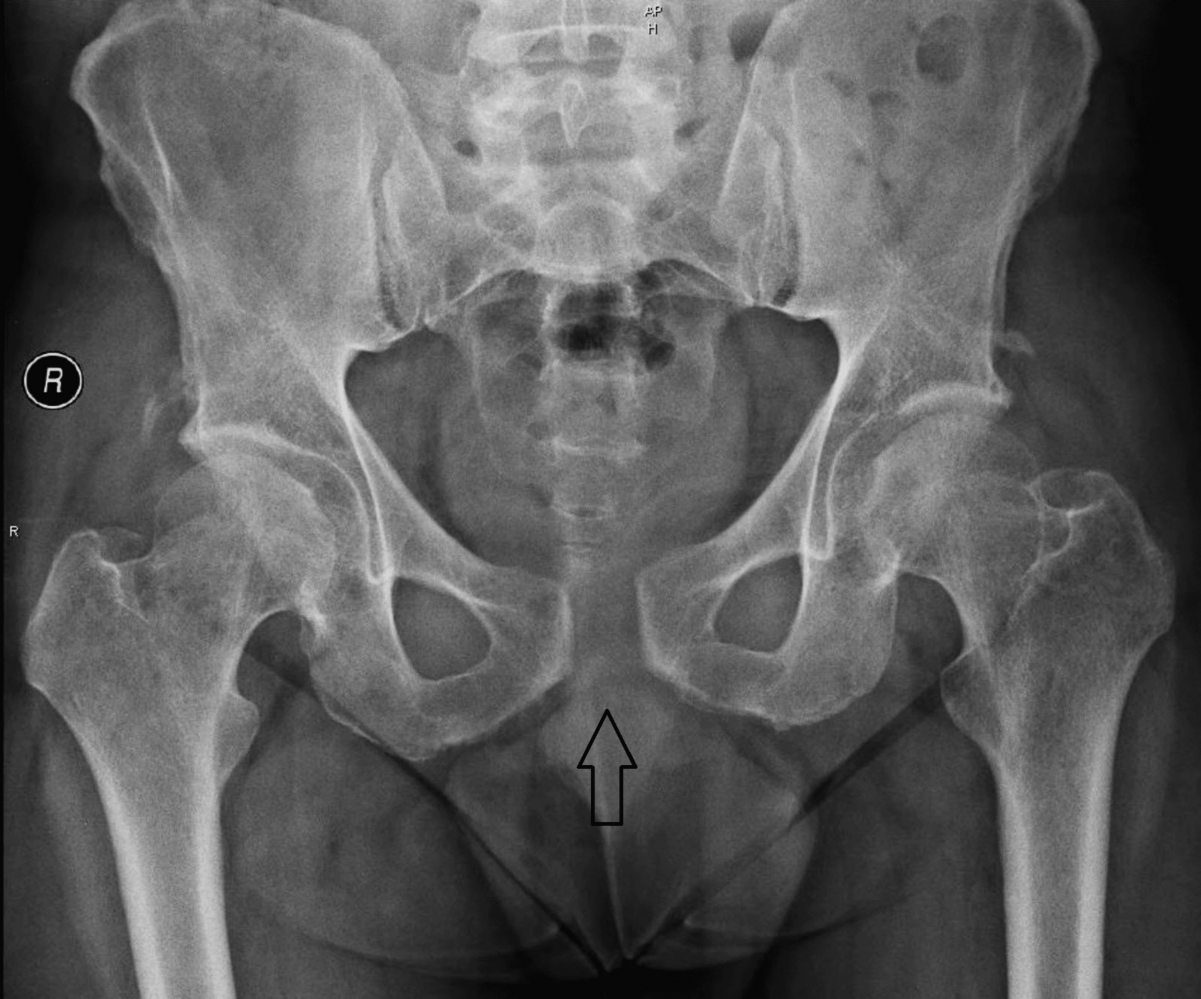
Anteroposterior radiograph of pelvis eight months after injury showing no further loss of reduction.

A full return to daily routine was conducted at 4 months from the injury without any discomfort and to pre-injury sports-related activities after completing six months of rehabilitation physiotherapy that focused on muscle strength, proprioception, and flexibility. He was lastly followed up at our department at eight months post-injury, where the x-ray showed no significant loss of reduction (1.9 cm gap) (Figure 5).

## Discussion

Pelvic damage after horse-related activities mostly occurs when the rider falls from the horse [8,11]. In case of massive retroperitoneal hemorrhage with injuries to the urethra, bladder, or rectum that could be fatal, early intervention, including resuscitation, control, and management of bleeding, could be life-saving [12]. The nature of the injury often depends on the rider’s position at the time of impact, the direction of the force applied, and whether the force is direct. This can result in lateral compression injuries, anteroposterior injuries, or a combination of both [11]. In addition, occasionally, when a horse rider is thrown clear of the horse, he strikes the ground forcibly and this mechanism of injury might result in a fracture of the acetabulum, which in rare cases might be accompanied by dislocation of the hip [13].

Nevertheless, in our case, the patient did not report any injury. The mechanism that led to the diastasis of the pubic symphysis could be related to the pommel of the saddle that split the symphysis [8,9]. Indeed, apart from direct trauma, other mechanisms may be involved in such occasions, including repeated strain from intense horse riding or improper riding technique, amongst others (Table 1) [7-9,11,13-15].

**Table 1:** Potential causes/mechanisms of diastasis of the pubic symphysis following horse riding.

Cause	Description of mechanism
Direct trauma	High-impact fall during horse riding may cause diastasis of the pubic symphysis.
Pelvic strain and overuse	Repeated strain from intense riding may lead to ligament laxity or separation over time (Repetitive micro-trauma in the saddle).
Weakness in pelvic ligaments	Pre-existing ligament laxity may increase the risk of diastasis when exposed to riding forces.
Prior pregnancy	Hormonal and structural changes may weaken the pubic symphysis, increasing susceptibility to separation after horse riding.
Improper riding technique	Poor riding posture or technique may put additional stress on the pelvis, contributing to diastasis risk.

In terms of pelvic biomechanics, the post-acetabular region of the pelvis is regarded as an arch, with the sacrum as its keystone [9]. The area in front of the acetabulum serves as a stabilizing "tie" for this structure [9]. The pubic symphysis and sacroiliac joints allow each hip bone to perform a slight lateral swing, restricted by the lower sacroiliac ligaments and the pubic symphysis’s arcuate ligament [9]. This movement acts as a buffer, absorbing moderate forces transmitted to the pelvis, such as those encountered in horseback riding [9]. However, Flynn described that as the pubic arch is brought down on the anterior part (pommel) of the saddle, which is often a rounded wedge, this wedge splits the pubic arch, causing a wider separation of the hips than normal [9]. Possibly in our patient, while horse riding, vertical stresses were applied to the pelvis from the pommel repeatedly, which caused gradual diastasis of the pubic symphysis. Thus, he did not mention any noticeable injury when he was admitted to our Hospital, such as a violent landing astride the saddle's pommel after horse bucking.

In the literature, only infrequent events of riders with a similar mechanism of pelvic injury are reported. Mulhall et al. demonstrated in a case series article three cases of pelvic injury that were treated operatively with either an external fixator or internal fixation with dynamic compression plate and screws [8]. The first two patients were recreational horse riders, and the third one was a professional jockey. On the first occasion, the rider was injured when the horse bucked several times and landed violently on the pommel of the saddle, presenting post-traumatic extensive scrotal and perineal bruising. The second one also displayed extensive bruising in the anterior part of the perineum when he struck the pommel as the horse landed after jumping over a fence. The third patient, while galloping on a horse, lost control and landed violently two or three times on the pommel before falling off. In contrast to our patient, the typical pattern of bruising was also noticed here, as in the other aforementioned patients [8].

In parallel, Flynn reported two cases of patients who suffered from disruption of the symphysis pubis after landing astride the pommel of the saddle while horse riding. Both patients threw themselves off the horse to the ground with severe pain. Bruising and discoloration in the region of the pubic symphysis were also observed [9]. In opposition to our rider, these patients were treated nonoperatively with the application of a pelvic sling.

Regarding the diagnosis, physical examination and imaging assessment were crucial for early diagnosis and successful management of our patient. Despite the absence of noisy symptomatology for such severe damage, the radiographs revealed a pubic symphysis diastasis of more than 3 cm that required fixation without posterior instability, as confirmed in the CT scan. The external fixation that was applied offered easy and fast stabilization of the anterior instability. The light widening of the pubic symphysis that was noticed when the patient was advised for full weight bearing 7 weeks after surgery was managed with the maintenance of the external fixator for 3 more weeks, and no further adjustments were required.

## Conclusions

Although horse riders are mostly young and healthy, horse-related injuries should not be underestimated. Pelvic ring injury in such patients might occur not only after a fall from a horse but also after atypical injury patterns, and thus, they might be misdiagnosed. Thus, clinicians should always consider pubic symphysis diastasis in patients presenting with sudden onset of localized pain and difficulty walking after horseback riding, even if there is no history of trauma or typical bruising in the pubic area. In addition, an effective diagnostic approach is crucial for successful management in such cases.
